# Drug coverage in treatment of malaria and the consequences for resistance evolution - evidence from the use of sulphadoxine/pyrimethamine

**DOI:** 10.1186/1475-2875-9-190

**Published:** 2010-07-05

**Authors:** Allen L Malisa, Richard J Pearce, Salim Abdulla, Hassan Mshinda, Patrick S Kachur, Peter Bloland, Cally Roper

**Affiliations:** 1 Sokoine University of Agriculture, Department of Biological Sciences, Faculty of Science, SUA, PO Box 3038, Morogoro, Tanzania; 2 Ifakara Health Institute (IHI), PO Box 53, Ifakara, Kilombero District, Tanzania; 3 London School of Hygiene and Tropical Medicine, Pathogen Molecular Biology Unit, Department of Infectious Tropical Diseases, Keppel Street, London, WC1E 7HT, UK; 4 Malaria Branch, Division of Parasitic Diseases, National Center for Infectious Diseases, Centers for Disease Control and Prevention (CDC), Atlanta, GA, USA

## Abstract

**Background:**

It is argued that, the efficacy of anti-malarials could be prolonged through policy-mediated reductions in drug pressure, but gathering evidence of the relationship between policy, treatment practice, drug pressure and the evolution of resistance in the field is challenging. Mathematical models indicate that drug coverage is the primary determinant of drug pressure and the driving force behind the evolution of drug resistance. These models show that where the basis of resistance is multigenic, the effects of selection can be moderated by high recombination rates, which disrupt the associations between co-selected resistance genes.

**Methods:**

To test these predictions, *dhfr *and *dhps *frequency changes were measured during 2000-2001 while SP was the second-line treatment and contrasted these with changes during 2001-2002 when SP was used for first-line therapy. Annual cross sectional community surveys carried out before, during and after the policy switch in 2001 were used to collect samples. Genetic analysis of SP resistance genes was carried out on 4,950 *Plasmodium falciparum *infections and the selection pressure under the two policies compared.

**Results:**

The influence of policy on the parasite reservoir was profound. The frequency of *dhfr *and *dhps *resistance alleles did not change significantly while SP was the recommended second-line treatment, but highly significant changes occurred during the subsequent year after the switch to first line SP. The frequency of the triple mutant *dhfr *(N51I,C59R,S108N) allele (conferring pyrimethamine resistance) increased by 37% - 63% and the frequency of the double A437G, K540E mutant *dhps *allele (conferring sulphadoxine resistance) increased 200%-300%. A strong association between these unlinked alleles also emerged, confirming that they are co-selected by SP.

**Conclusion:**

The national policy change brought about a shift in treatment practice and the resulting increase in coverage had a substantial impact on drug pressure. The selection applied by first-line use is strong enough to overcome recombination pressure and create significant linkage disequilibrium between the unlinked genetic determinants of pyrimethamine and sulphadoxine resistance, showing that recombination is no barrier to the emergence of resistance to combination treatments when they are used as the first-line malaria therapy.

## Background

In a new era of anti-malarial treatment it is important to examine the use of past drugs and to determine which drug use practices are most likely to preserve drug efficacy and to examine their specific importance across a range of malaria endemicity settings. High recombination rates associated with high malaria transmission found in much of sub-Saharan Africa [1] have the potential to delay the emergence of resistance by disrupting associations between the multigenic components of resistance [2-4]. This consideration is important when resistance is multigenic, as is the case with chloroquine, quinine [5,6] and SP [7], and will certainly be the case with the new artemisinin-based combination therapy (ACT). Mathematical models indicate that drug coverage is the primary determinant of drug pressure and the driving force behind the evolution of drug resistance [3,4]. These models show that where the basis of resistance is multigenic, the effects of selection can be moderated by high recombination rates, which disrupt the associations between co-selected resistance genes. In this study, the interplay between drug coverage, and the frequency of resistance genes in *Plasmodium falciparum *populations were examined in a highly endemic region of Africa [1]. Genetic changes occurring in the parasite reservoir of two rural sites in Tanzania were measured over a period of transition from low SP coverage while chloroquine was first-line treatment of uncomplicated malaria to higher coverage after SP became the first-line treatment.

The genetic determinants of pyrimethamine resistance are point mutations at codons 16, 50, 51, 59, 108 and 164 of the *dhfr *gene [8,9]. Genetic determinants of sulphadoxine resistance are mutations found at codons 436, 437, 540, 581 and 613 of the *dhps *gene [10,11]. In African *P. falciparum*, the presence of three *dhfr *mutations (N51I, C59R, S108N) together with two *dhps *mutations (A437G, K540E) prior to treatment is a significant predictor of SP treatment failure [12-14]. In southeast Africa, pyrimethamine resistance became established prior to sulphadoxine resistance. This was evidenced by *in-vitro *studies in Kenya, which showed that pyrimethamine resistance was common as early as 1988, while sulphadoxine resistance did not appear until 1993-1995 [15]. Molecular genetic studies have corroborated the *in-vitro *observations, showing that double mutant *dhfr *was present in Kisumu, Kenya at least as early as 1981 [16], while resistant *dhps *only appeared between 1993 and 1995 [17]. The emergence of the *dhps *double mutant (A437G K540E) on a background of resistant *dhfr *coincided with the emergence of clinically evident SP resistance in Africa. The *dhps *double mutant (A437G, K540E) was first recorded in Kenya between 1993 and 1995 [17], Tanzania in 1995 [18], and it was first seen in Malawi in 1995/1996 [19]. In north-eastern Sudan, it was absent in 1993 but had appeared by 1998 [20], and in KwaZulu Natal, South Africa, it was absent in 1995/1996 but had appeared by 1999 [21]. Analysis of microsatellite polymorphisms around resistant *dhfr *and *dhps *suggests that underlying these events was a dispersal of the *dhps *double mutant allele on a background of resistant *dhfr *throughout eastern and southern Africa [21].

Treatment of malaria in Tanzania is typically guided by official recommendations from the Ministry of Health and Social Welfare regarding drugs of choice for various situations. "First-line" treatment refers to the drug officially recommended as the drug of first choice for the treatment of uncomplicated malaria. "Second-line" treatment refers to the drug officially recommended as an alternative primarily to be used for treatment of patients in whom the first line treatment failed to clear the infection and other select patients. "Third-line" treatment typically refers to the drug recommended for severely ill patients. In practice, few treatment failures are recognized and patients are often moved directly from first to third line treatment, consequently, little second line drug is used compared to the first line drug. SP replaced chloroquine as the recommended first line treatment on the Tanzanian mainland in August 2001. Prior to that SP was the recommended second line treatment. A survey of SP efficacy prior to national policy change showed a marked regional variability within Tanzania with clinical efficacy ranging between 76% in Tanga and 93% in Mwanza [22]. Tanga, in north-east Tanzania, was the first site in Africa where SP treatment failure was reported [23,24]. Our investigation took place in Kilombero/Ulanga and Rufiji Districts which are located > 400 km from Tanga and where resistance has been slower to appear. A study among children aged 6-59 months in Kilombero during 1999 found parasitological clearance was 94% over 28 days of follow-up after SP treatment [25].

To examine the genetic change at *dhfr *and *dhps *loci under first and second line SP use in Kilombero/Ulanga and Rufiji Districts, malaria parasite samples were collected from residents of randomly selected households in the two districts, in a series of cross sectional surveys, over an area covering 29,400 km^2 ^and including sites up to 300 km apart. Three cross sectional surveys were performed. The first survey occurred during July-September 2000, when CQ was first-line treatment and SP was second line. The second survey in July -September 2001 coincided with the implementation of a new national policy recommending use of SP as the first-line treatment of uncomplicated malaria. The third survey, in July-September 2002, occurred approximately one year after implementation of that policy change. In all, the surveys included 20,062 participants and yielded parasites from 4,950 malaria infections. By comparing genetic change between 2000 and 2001 with that which occurred between 2001 and 2002, the impact of the policy change on the genetic composition of the parasite population was quantified. Infections identified at community level include both symptomatic and asymptomatic participants. This method of sampling is preferable to sampling at a health clinic where by definition all infections are symptomatic, and many might have been pretreated. Since resistant parasites are selected by pre-treatment, the active surveillance approach yields a less biased sample of the circulating parasite population at community level.

## Methods

### Study area, subjects and samples

Community surveys were conducted during July, August and September of 2000, 2001 and 2002 in three rural districts of south-eastern Tanzania, Rufiji (Population = 170,000), Kilombero (Population = 220,000) and Ulanga (Population = 160,000). The three districts were well matched in terms of predicted intensity and duration of malaria transmission and risk (MARA), relative access and overall utilization of health services (based on surveys), fairly usage of insecticide treated nets (ITNs) and relative proportion of urban peri-urban, rural population. The surveys were part of large combination therapy pilot implementation programme in Tanzania, the Interdisciplinary Monitoring Programme for Antimalarial Combination Therapy (IMPACT-TZ). IMPACT-Tanzania is a multiyear implementation research evaluation that rests on a collaborative platform incorporating the US Center for Disease Control and Prevention (CDC), the Ifakara Health Institute, London School of Hygiene and Tropical Medicine, and the Ministry of Health and Social Welfare including its National Malaria Control Programme, the Tanzania Essential Health Interventions Project and the Council Health Management Teams of Rufiji, Kilombero, Ulanga, Morogoro and Mvomero Districts. IMPACT-Tanzania is primarily supported by funding from the United States Agency for International Development, CDC and Wellcome Trust. For the purpose of the study, Kilombero and Ulanga Districts were treated as a single district because population movement between these two districts is high and the study population spans the border region. *Plasmodium falciparum *malaria transmission in the study area is intense (with an estimated entomological inoculation rate of 367 infectious bites per person per year [1]) and perennial with some seasonal fluctuation. A total of 20,062 adults and children belonging to randomly selected households participated in the study. A finger-prick blood sample for blood slide and filter paper bloodspot were collected from each individual in the household. The filter paper bloodspots were air-dried and stored at room temperature in self-sealing plastic bags with dessicant and stored dry at room temperature. All blood slide samples were screened by light microscopy for *P. falciparum *parasites. Bloodspots from microscopically positive subjects were selected and preserved at room temperature for molecular genotyping.

### Ethics

Scientific and ethical clearance was granted from the Medical Research Council of the National Institute for Medical Research in Tanzania, the Centers for Disease Control and Prevention, USA, and the London School of Hygiene and Tropical Medicine. Written informed consent was obtained from all individuals or their guardians before collection of samples.

### DNA extraction

The DNA was extracted from bloodspots dried on filter papers. A section of the dried blood spot filter paper was excised using a sterile blade or scissors, and soaked in a 1 ml, 0.5% saponin-1× phosphate buffered saline (PBS) overnight in a 96-deepwell plate. The section of filter paper was then washed twice in 1 ml of 1× PBS and finally, was boiled for 8 min in 100 μl PCR quality water with 50 μl 20% chelex suspension (pH 9.5).

### PCR amplification

Nested PCR was used to amplify a 594 base pair (bp) fragment of *dhfr *and a 711 bp fragment of *dhps *each containing the sequence where mutations are found. Primer sequences and PCR reaction conditions were previously described in [25]. PCR was performed in 96 well plates with 25-μl PCR reaction volumes containing final concentrations of 0.25 μM oligonucleotide primers, 2 mM MgCl_2_, 250 μM each deoxyribonucleotide triphosphate (dNTPs), and 1× Taq polymerase. 1 μl of DNA template was used in the outer (primary) PCR reaction mixture for *dhfr *and *dhps *amplifications. For the inner (secondary) *dhps *reactions 1 μl of the outer PCR product was used. The outer *dhfr *PCR products were diluted three fold before 1 μl was introduced into the inner PCR reaction mixtures.

### Molecular genotyping of point mutations by sequence specific oligonucleotide probing (SSOP)

The amplified PCR products were screened for *dhfr *and *dhps *sequence variants at 10 loci where single nucleotide polymorphisms (SNPs) related to anti-malarial drug resistance are known to occur. The sequence changes (and the amino acid substitutions they code for) are summarized in Table 1.

**Table 1:** The nucleotide and amino acid substitutions at (a) dhfr and (b) dhps genes screened for by PCR-SSOP

dhfr					
Codon	50	51	59	108	164

Wild type	Cys (C) TGT	Asn (N) AAT	Cys (C) TGT	Ser (S) AGC	Ile (I) ATA
		AAC			

Mutant	**Arg (R)**	**Ile (I)**	**Arg (R)**	**Asn (N)**	**Leu (L)**
	**CGT**	**ATT**	**CGT**	**AAC**	**TTA**
				**Thr (T)**	
				**ACC**	

dhps					

Codon	436	437	540	581	613


Wild type	Ser (S)	Ala (A)	Lys (K)	Ala (A)	Ala (A)
	TCT	GCT	AAA	GCG	GCC

Mutant	**Phe (F)**	**Gly (G)**	**Glu (E)**	**Gly (G)**	**Ser (S)**
	**TTT**	**GGT**	**GAA**	**GGG**	**TCC**
	**Ala (A)**				**Thr (T)**
	**GCT**				**ACC**
	**Cys (C)**				
	**TGT**				

PCR products were spotted in a 12 by 8-grid and cross linked onto nylon membranes and probed for sequence polymorphisms by hybridization to specific oligonucleotide probes described previously [26]. For analysis of samples collected in 2000, the visualization of hybridized digoxygenin labelled probes on membranes was performed by the alkaline phosphatase-catalysed breakdown of the CSPD substrate (Roche Boehringer Mannheim, Mannheim, Germany) and visualized by exposure on Hyperfilm-ECL (Amersham Pharmacia Biotech, Little Chalfont, Buckinghamshire, United Kingdom), according to Boehringer Mannheim recommendations and previously described [27]. For analysis of samples collected in 2001 and 2002 the probed blots were visualized using ECF substrate and detection using a phosphoimager (STORM^®^). Inspection of autoradiographic films was carried out by light box illumination, while the phophoimager output was recorded through viewing of digitally-captured images of chemifluorescent signal. The change in the method by which probe hybridization signal was visualized did not affect the results in any way since the probes and hybridization conditions were unchanged.

The stringency and specificity of the hybridization process was confirmed by inspection of a series of 4 controls with a known single genotype variant sequence. All blots with non-specifically bound probes were stripped and re-probed. A SNP was considered to be present in the PCR product when the intensity of signal was higher than that of the background. The blots were scored independently by two people.

In this analysis, the aim was to establish the relative abundance of different point mutation haplotypes at *dhfr *and *dhps*. Since bloodstage *P. falciparum *is haploid, this is very straightforward when an infection consists of a single genotype because only one form of sequence at every SNP locus is seen. When infections are composed of multiple genotypes a mixture of different sequence variants occurs making the inference of point mutation haplotypes within that infection more difficult.

The presence, absence, and relative abundance of hybridization signal for every probe were recorded at each locus. A sample was considered to have a single haplotype when only one sequence variant was found at each locus. Blood samples were categorized as having a single, a majority, or a mixture of sequence at each SNP locus. Majority and mixed genotype infections were differentiated according to the relative intensity of signal. If the hybridization signal of the minority sequence was greater than half the intensity of the majority then an infection was classified as mixed. To determine the relative abundance of different point mutation haplotypes in the parasite population, one haplotype only was counted from each infection and those mixed infections where haplotypes could not be resolved were omitted from the calculation of haplotype frequencies. Hence, frequency data is based upon a subset of isolates which were either unmixed or had a predominating majority haplotype. A breakdown of the proportions of isolates which successfully PCR amplified and which were genotyped as single, majority or mixed haplotype infections is given in Table 2.

**Table 2:** Annual survey 2000-2002, malaria positive samples and their PCR outcome in Rufiji and Kilombero/Ulanga populations.

	Rufiji			Kilombero Ulanga		
**Year**	**2000**	**2001**	**2002**	**2000**	**2001**	**2002**

Survey population	2844	3285	3349	3289	3197	4098

*P. falciparum *positive	778	908	854	955	580	875

PCR amplified *dhfr*	549	683	687	404	488	686

PCR amplified *dhps*	521	592	725	444	347	720

Single or majority *dhfr*	455	420	527	376	238	489
	83%	62%	77%	93%	49%	71%

Single or majority *dhps*	417	519	596	365	294	603
	80%	88%	82%	82%	85%	84%

Single or majority *dhfr+dhps*	288	278	404	190	138	381
	55%	47%	59%	47%	40%	55%

### Statistical analysis

Statistical comparison of allele frequencies at *dhfr *and *dhps *in the various sites was carried out using chi-squared analysis in STATA version 9.2 [28]. The calculation of binomial exact 95% confidence intervals was carried out using STATA version 9.2. Linkage disequilibrium analysis was performed using Arlequin software.

### Role of the funding source

This study was funded through an interagency agreement between the United States Agency for International Development (USAID) and CDC and a cooperative agreement between CDC and the Ifakara Health Institute (IHI). USAID did not participate in the design, collection, analysis, or interpretation of the data, in the writing of the report, or in the decision to submit for publication.

## Results

Of 20,062 people sampled, 4,950 were identified as infected with *P. falciparum*. DNA was extracted from the 4,950 *P. falciparum *positive bloodspots and PCR amplification of *dhfr *and *dhps *performed once, giving a combined rate of PCR amplification success of 69% for both genes (Table 2). The amplified products were screened for all the variant sequences described in Table 1. Out of the 3,436 isolates, which amplified successfully for *dhfr*, 71% were single or majority genotype infections and the point mutation haplotypes could easily be resolved. Of the 3,412 samples, which amplified successfully for *dhps*, 81% were single or majority genotype with resolvable haplotypes.

### Allelic haplotypes at *dhfr *and *dhps *genes

The point mutations found in the *dhfr *gene were N51I, C59R and S108N. They were found in the following haplotypic arrangements NCS, NC**N **,N**RN**, **I**C**N **and **IRN **which are common throughout East Africa and have been previously reported in Tanzania [26], Malawi [29], Kenya [12,30], and Uganda [31,32]. Two rare combinations of mutations were found; N51I with C59R (**IR**S) was found in a single individual (previously reported in Uganda [31], and C59R alone (N**R**S) was found in one sample only and has not been previously reported, (again sequence confirmation of these rare haplotypes needs to be done).

Five *dhps *mutations were found (S436A, S436F, S436C, A437G, and K540E) in nine distinct haplotypic arrangements; five of which (SAK, **A**AK, S**GE **,S**G**K, and SA**E **) have been described previously in isolates from East Africa [12,26,30-32] while the remaining four (**C**AK, **F**AK, **A**A**E **,and **F**A**E**) were found in extremely low frequency and have not been reported before, presumably because of their rarity, although sequencing confirmation is also needed to verify their existence.

**Figure 1:**
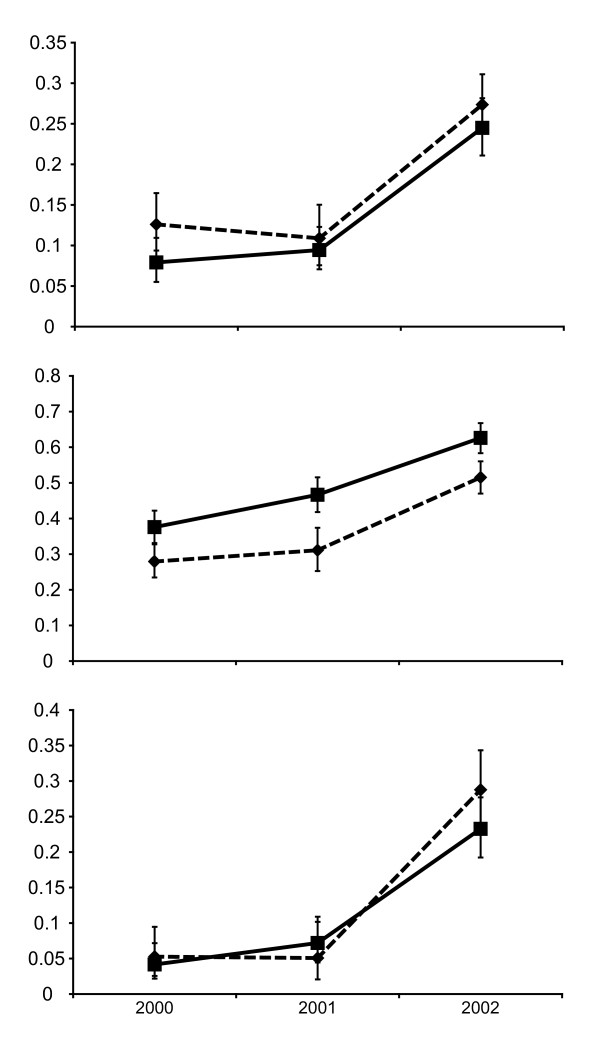
**Changes in the frequency of SP resistance genes in Kilombero/Ulanga (dotted line with diamonds) and Rufiji (solid line with squares) in cross sectional surveys in 2000, 2001, and 2002**. Top graph is the frequency of the *dhps *double mutant allele, middle graph the frequency of *dhfr *triple mutant alleles and the bottom graph the frequency of triple mutant *dhfr *+ double mutant *dhps *genotype.

The change in frequency of the *dhfr ***IRN **and the *dhps *S**GE **haplotypes, which have the greatest significance for SP efficacy are shown in Figures 1a and 1b. Changes occurring under the CQ policy (2000 to 2001) are markedly different to those occurring under the SP policy (2001-2002). The frequency of the *dhps *double mutant A437G K540E, shown in Figure 1a, did not change significantly between 2000 and 2001 in either Rufiji (0.08-0.09) (p > 0.411, 95% CI) or in Kilombero/Ulanga (0.13-0.11) (p > 0.497, 95% CI). Contrastingly, there was a highly significant change between 2001 and 2002 in both Rufiji (0.09-0.25) (2.5 fold increase p ≤ 0.0001, 95% CI) and Kilombero/Ulanga (0.11-0.27) (2.6 fold increase p ≤ 0.0001). The frequency of the *dhps *double mutant haplotype was remarkably similar in Kilombero/Ulanga and Rufiji and there were no significant differences between the two sampling sites at any time point.

The change in frequency of the *dhfr ***IRN **and the *dhps *S**GE **haplotypes, which have the greatest significance for SP efficacy are shown in Figures 1a and 1b. Changes occurring under the CQ policy (2000 to 2001) are markedly different to those occurring under the SP policy (2001-2002). The frequency of the *dhps *double mutant A437G K540E, shown in Figure 1a, did not change significantly between 2000 and 2001 in either Rufiji (0.08-0.09) (p > 0.411, 95% CI) or in Kilombero/Ulanga (0.13-0.11) (p > 0.497, 95% CI). Contrastingly, there was a highly significant change between 2001 and 2002 in both Rufiji (0.09-0.25) (2.5 fold increase p ≤ 0.0001, 95% CI) and Kilombero/Ulanga (0.11-0.27) (2.6 fold increase p ≤ 0.0001). The frequency of the *dhps *double mutant haplotype was remarkably similar in Kilombero/Ulanga and Rufiji and there were no significant differences between the two sampling sites at any time point.

Changes in the frequency of the *dhfr *triple mutant allele **IRN **are shown in Figure 1b. The frequency was significantly higher in Rufiji than in Kilombero/Ulanga at all time points. Between 2000 and 2001, there was an increase in frequency in both Kilombero/Ulanga (0.28-0.31) (p > 0.400, 95% CI) and Rufiji (0.38-0.47) (p < 0.007, 95% CI), yet between 2001 and 2002 the increase was greater and more highly significant in both Kilombero/Ulanga (0.31-0.52) (p ≤ 0.0001, 95% CI) and Rufiji (0.47-0.63) (p ≤ 0.0001, 95% CI).

In a further subset of samples where both *dhfr *and *dhps *sequences were unmixed, it was possible to measure the frequency of two locus genotypes. In figure 1c, the frequency of the triple *dhfr *+ double *dhps *genotype in the two populations is compared. The initial frequency was around 0.05 in both the districts and there was no change between 2000 and 2001 in Kilombero/Ulanga (p > 0.824, 95% CI) or Rufiji (p > 0.186, 95% CI) but a remarkable 4 fold increase to frequencies of 0.21 and 0.24 occurred between 2001 and 2002 in Kilombero/Ulanga (p < 0.0001, 95% CI) and Rufiji (p < 0.0001, 95% CI).

### Haplotypes conferring intermediate levels of resistance

**Figure 2:**
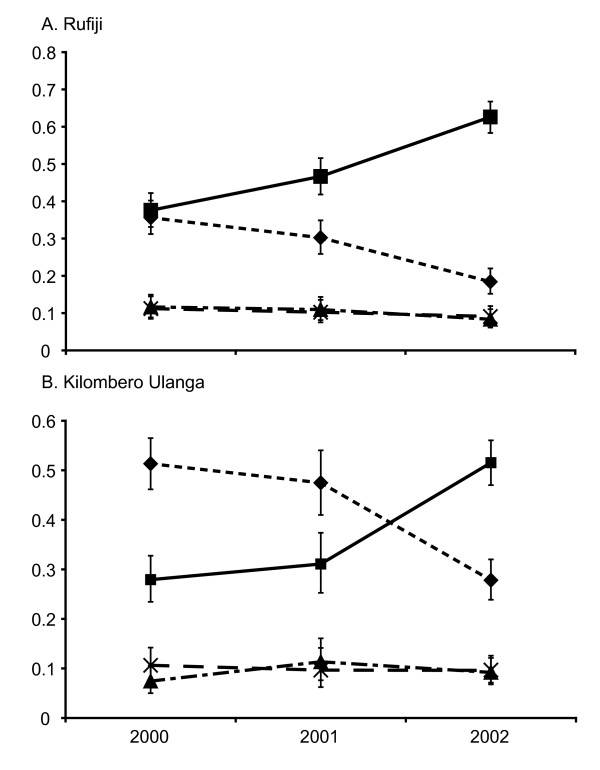
**Allele frequency changes at *dhfr *in Rufiji (A), and Kilombero/Ulanga (B)**. The sensitive allele (dotted line with diamond), the triple mutant N51I + C59R + S108N allele (solid line with squares), and double mutant C59R + S108N (dash-dot line with triangles), double mutant N51I + S108N(dashed line with X).

The effect of changing policy on the frequency of sensitive and double mutant *dhfr *alleles is shown in Figure 2. The increase of the triple mutant allele acted to displace sensitive alleles, which show a substantial decline in Kilombero/Ulanga 2000-2001 (0.51-0.47) (p > 0.352, 95% CI), 2001-2002 (0.47-0.28) (p ≤ 0.0001, 95% CI) and Rufiji 2000-2001 (0.36-0.30) (p > 0.092, 95% CI) and 2001-2002 (0.30-0.18) (p ≤ 0.0001, 95% CI). The double mutant *dhfr *alleles, which confer intermediate levels of resistance *in-vitro *[33], neither increased nor decreased, remaining at a frequency of around 10% in both sites throughout all surveys.

**Figure 3:**
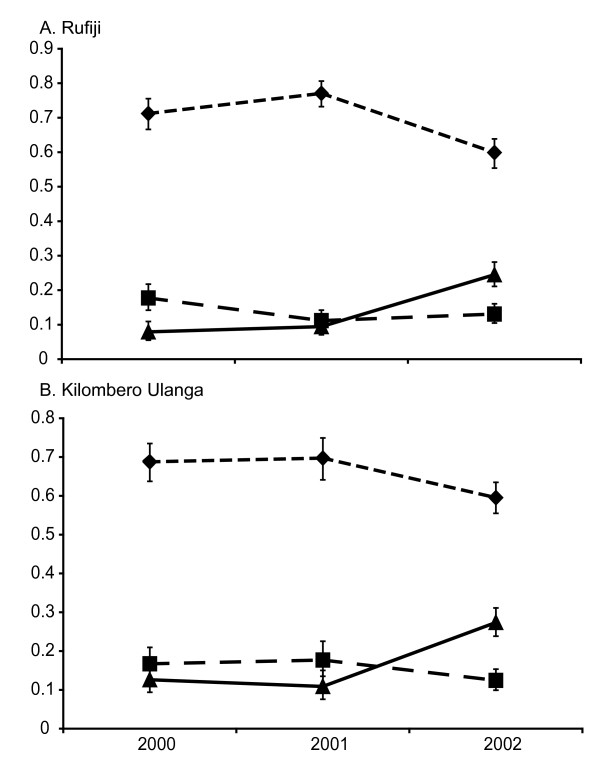
**Allele frequency changes at *dhps *in Rufiji (A), and Kilombero/Ulanga (B)**. The sensitive allele (dotted line with diamonds), the double mutant A437G + K540E allele (solid line with triangles), and single S436A mutant (dashed line with squares).

As well as the highly resistant *dhps *A437G K540E double mutant, a number of single 436 mutant alleles were recorded. Among these, by far the most common was the S436A, which was consistently found at frequencies of 10%-20% at all time points in both districts. Figure 3 shows the frequencies of the sensitive S436A single mutant and the A437G K540E double mutant alleles through time in both districts. From these data it is clear that the rising frequency of the double mutant allele displaced the sensitive allele, which decreased significantly during 2001-2002 in both Kilombero/Ulanga (0.7-0.6) (p ≤ 0.001, 95% CI) and Rufiji (0.77-0.60) (p ≤ 0.0001, 95% CI). Interestingly, the frequency of the A436A allele remained static at all time points in both sites.

### Linkage disequilibrium

**Figure 4:**
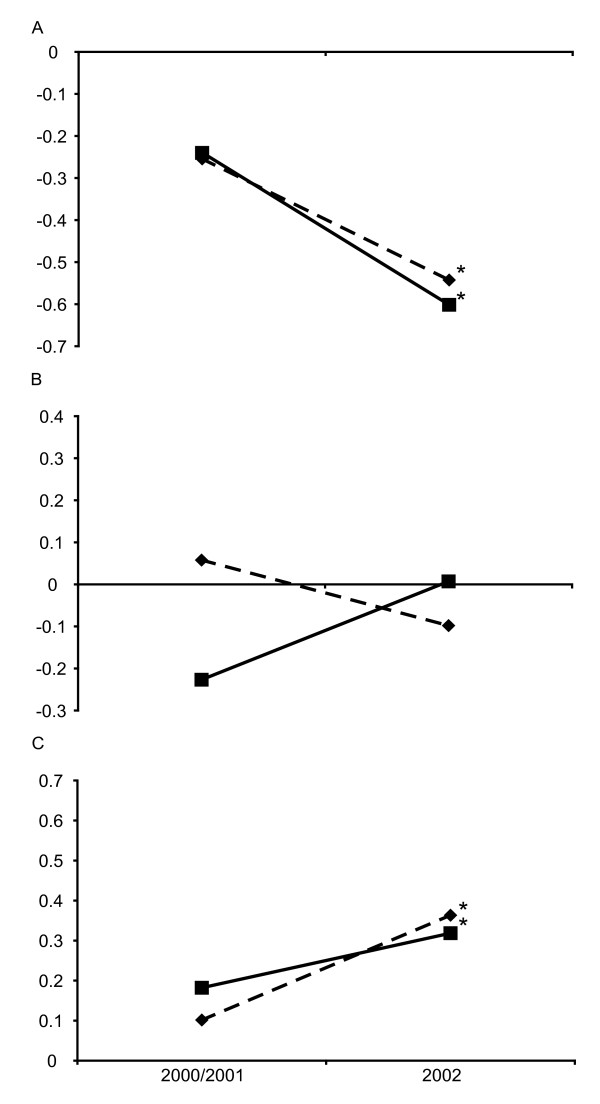
**Linkage disequilibrium between the *dhps *A437G + K540E allele and different alleles at *dhfr *(A) the *dhfr *triple mutant N51I + C59R + S108N (B) *dhfr *double mutants C59R + S108N and N51I + S108N (C) the *dhfr *sensitive allele**. The d' values for 2000 + 2001(Kilombero/Ulanga n = 328, Rufiji n = 566) combined and for 2002 (Kilombero/Ulanga n = 381, Rufiji n = 404) are shown. Significant deviation between observed from expected occurred in 2002 indicated by *(p < or = 0.001).

To examine the effect of simultaneous selection by pyrimethamine on *dhfr *and sulphadoxine on *dhps*, we looked at two-locus genotypes sampled from both sites in the three successive surveys. Taking the subset of samples for which point mutation haplotypes could be unequivocally resolved for both genes, we compared the observed with expected frequencies generated from contingency tables (given in full in Additional file 1). No significant linkage disequilibrium was found between *dhfr *and *dhps *loci in either 2000 or 2001. Since these surveys were both conducted while SP was second-line therapy, the data was pooled and, in Figure 4, linkage disequilibrium (d') is compared under second line SP selection (2000 and 2001 combined) and during first line SP selection (in the 2002 survey). There was a significant association between the *dhfr *triple mutant allele and the *dhps *double mutant allele after the change in policy. In Kilombero/Ulanga during 2000-2001, 17 of the 328 unmixed samples carried the *dhfr *triple *dhps *double mutant genotype (d' = 0.1015, p > 0.2885, 95% CI) while in Rufiji 2000-2001, 32 of 566 unmixed samples had the *dhfr *triple *dhps *double mutant genotype (d' = 0.1821, p > 0.0760, 95% CI). By 2002 there was a highly significant association between these alleles. In Kilombero/Ulanga 84 of 381 unmixed samples carried the *dhfr *triple *dhps *double mutant genotype and 64 were 'expected' (d' = 0.3633, p ≤ 0.0001, 95% CI) and in Rufiji 94 of 404 unmixed samples carried this same genotype compared to 79 'expected' (d' = 0.3184, p ≤ 0.001, 95% CI). The d' index of linkage disequilibrium, between the *dhps *A437G + K540E double mutant and *dhfr *double mutant allele was not significantly changed (Figure 4B) but a negative association between the *dhps *double mutant allele and *dhfr *sensitives emerged under first line SP treatment (Figure 4C) (Rufiji p ≤ 0.0001, 95% CI, and Kilombero/Ulanga p ≤ 0.0001, 95% CI).

### Measurement of haplotype frequencies

Measurement of haplotype frequencies is complicated by the existence of mixed infections. If multiple haplotypes are counted from mixed infections the frequency of rare allelic haplotypes is overestimated. Haplotype frequency measures presented were based solely on infections where a single or majority haplotype was detected, and only one haplotype was scored per individual infection, while mixed infections were excluded because point mutation haplotypes could not be determined. The overall proportion of mixed infections excluded from the frequency estimations was 29% of infections for *dhfr *and 19% of infections for *dhps*.

**Figure 5:**
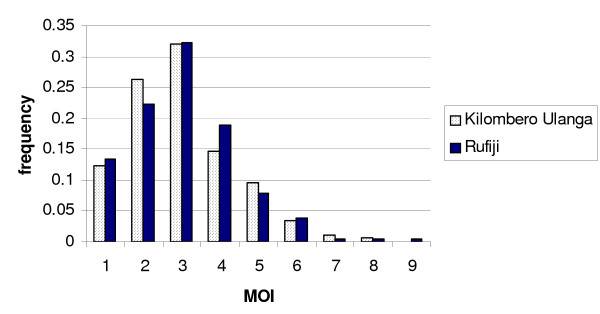
**The minimum number of co-infecting genotypes (multiplicity of infection or MOI) was determined by measuring the number of alleles in every sample at 3 unlinked microsatellite loci (Poly A, Pfpk2 and TA109)**. Here the MOI in 178 samples from Kilombero/Ulanga 2002 (white) and 180 samples Rufiji 2002 (black) are compared.

To examine the extent of the underlying rate of mixture in infections and test the robustness of this frequency estimates, this study looked at three unlinked microsatellite markers (Poly A, Pfpk2 and TA109) in 178 and 180 samples from 2002 survey from Kilombero/Ulanga and Rufiji, respectively. The minimum number of co-infecting genotypes in each infection was determined as the greatest number of alleles at any of the three microsatellite loci. This number is often referred to as the "multiplicity of infection" or (MOI). In Figure 5 the distribution of MOI in the two districts is compared showing that they are similar. The presence/absence of *dhps *A437G and K540E among the same infections was used to test whether the distribution of the dhps double mutant alleles was consistent with expectation using a method devised by Schneider *et al *[34] (see Additional file 1). This uses maximum likelihood to predict the underlying frequency of resistance alleles based on measures of the multiplicity of infection and the presence/absence of resistance mutations in those infections. The analysis predicted an underlying frequency of the *dhps *double mutant of 0.276 in Rufiji (where the frequency among single and majority genotype infections was 0.274) and a frequency of 0.262 in Kilombero Ulanga (where the frequency among single and majority genotype infections was 0.245). The estimates using these two approaches are very consistent showing that no obvious bias is introduced into the estimation of frequencies by excluding mixed infections.

The robustness of this approach for estimating allele frequencies in populations sampled by blood survey has been demonstrated in a study by Anderson *et al *[35] comparing three different methods. All three methods gave equivalent frequency values. The predominant allele adopted in our study is favoured because it utilizes data from as many individuals as possible while still giving reliable haplotype data. Importantly the MoI did not vary significantly between treatments at any time it therefore cannot be the source of any systematic bias in our analysis. Majority or minority, the genotypes important in the spread of resistance must, by definition, appear in greater frequency in subsequent surveys. The power of examining a series of cross sectional surveys is its capacity to show the significant micro-evolutionary genetic changes over time.

## Discussion

### Policy and genetic change

The influence of policy change on the genetic composition of the parasite population was profound. While the frequency of *dhfr *and *dhps *alleles did not change significantly during 12 months of first line CQ treatment and second line SP, the switch to a policy of first line SP caused the frequency of the triple mutant *dhfr *allele to increase by 37% to 63% and the frequency of the double mutant *dhps *allele to increase 200-300%. A combination of these two alleles is predictive of SP treatment failure *in-vivo *[12-14] and the rapid increase in the frequency of this genotype from 5.5% to 22.7% between 2001 and 2002 is consistent with findings of a similar study in Mozambique [36], yet suggests that the outlook for SP efficacy in subsequent years would be increasingly bad. In fact, this observation is borne out by the results of SP treatment efficacy monitoring in south-east Tanzania during 2003 which found that 49% of SP treatments failed by day 28 [37]. A national policy decision was made in 2006 to switch the recommended first-line treatment to artemether plus lumefantrine combination treatment.

Household interviews conducted simultaneously showed that there was a sharp increase in SP use after the policy transition (a 5-6 fold increase between 2000 and 2002). Prior to the introduction of SP as first line therapy SP was widely available for self-treatment via purchase from shops [38], as well as through formal health facilities as a second-line treatment. Although SP is currently being replaced as the recommended first-line treatment for malaria in Tanzania and much of the rest of Africa, it will continue to be used in intermittent preventive treatment of infants and pregnant women. The drug pressure applied when SP use is restricted to intermittent programmes of treatment in infants and pregnant women will more closely resemble the situation of 2000-2001. The predicted consequences of more restricted use of SP are, first, that the rate at which frequency of resistance alleles increase should decline, and second, that the association between the triple mutant *dhfr *allele and double mutant *dhps *allele maintained by high coverage drug selection will diminish. Both these predictions pre-suppose that the resistance alleles had not already become fixed in the population.

### The spread of resistance

For successful spread, a resistance mutant must be transmitted at a faster rate than the sensitive form. This occurs in the presence of drug pressure because of the differential survival and reproduction rates conferred by these alleles. The population-wide rates of change observed in the present study are attributable to changes in SP coverage during that period. In order to quantify drug pressure under a specific policy intervention, its effect upon the relative abundance, or frequency, of every haplotype needs to be measured because each allele is subject to a differing selection pressure by virtue of its differing resistance properties.

The finding that first line use of SP brought about the same genetic changes independently in Kilombero/Ulanga and in Rufiji indicates they are likely to have broad applicability elsewhere. But direct transposition to predict rates in other settings should take into account differences in access to drugs and in the infrastructure for provision of healthcare services that can differ substantially from country to country. Importantly, from a molecular genetic perspective, recent studies in west and central African countries still show the *dhps *A437G, K540E double mutant allele to be absent or very rare [39,33,40]. Only once these alleles are established will the rates of change observed here be predictive of change in such populations.

### Evolutionary history of resistance alleles

By following haplotype frequencies over time, the contrasting behaviors of mutant alleles which confer high, intermediate or mild resistance were described. The frequency of weakly resistant alleles such as the *dhfr *double mutant and the *dhps *single mutant did not respond to the increase in SP use. During the course of the three annual surveys, both *dhfr *double mutant alleles maintained a steady frequency of around 10%, and the *dhps *single mutant maintained a frequency of around 15%. They were neither displaced by the highly resistant alleles, as the sensitive alleles were, nor did they increase in frequency in response to elevated drug pressure.

Double mutant *dhfr *alleles are often regarded as precursors of the triple mutant since they can potentially convert to the highly resistant triple mutant allele by the simple acquisition of a single point mutation. Somewhat surprisingly, flanking microsatellite analysis has shown that the triple mutants in southeast Africa [21], and in the Kilombero/Ulanga site specifically [41], belong to a single lineage which originated in Asia [42]. The double mutant *dhfr *alleles found in this site belong to a restricted number of independently derived lineages [21], which are in all probability of African origin. So while the increase in frequency of triple mutant allele in Kilombero/Ulanga and Rufiji can be explained by the expansion of the Asian derived lineage under selection by SP treatment, the steady persistence of the mildly resistant double mutant *dhfr *lineages at around 10% requires some further explanation. The existence of conserved flanking region around them is evidence of selection [21] and there is further evidence that double mutant *dhfr *alleles can gain fitness advantage in specific circumstances. For example, they have been associated with treatment failure in individuals who have not had sufficient exposure to malaria to have acquired immunity [14,43] and while not affecting clinical efficacy in Colombia, the N51I + S108N double mutant was associated with prolonged parasite clearance times and with gametocytaemia 14 and 28 days after treatment, indicating they confer survival and reproductive advantages [44]. It has also been proposed that exposure to sub-therapeutic drug levels might select for weakly resistant, or drug tolerant parasites which are newly inoculated into people who recently received treatment [45].

An alternative explanation for the continuing existence of *dhfr *double mutant alleles is that they pre-date the use of SP. Pyrimethamine was used as monotherapy during the 1950s and 60s and numerous studies showed that resistance quickly arose locally in response to drug pressure [46-48]. Double mutant *dhfr *alleles could have been the basis of these early reports of pyrimethamine resistant malaria and may well have preceded the arrival of the *dhfr *triple mutant. Supporting evidence comes from studies in Kenya where double mutant alleles were in the majority in a panel of isolates collected as early as 19N51I + S108N; 21% and C59R + S108N; 47% (16). These reports are concurrent with measures of intermediate resistance of around 20% in Kilifi, coastal Kenya between 1984 and 1989. This rose to 92% between 1993 and 1995 [49], by which time the triple mutant was becoming more prevalent in Kilifi [50].

### The evolution of multi-drug resistance

By combining *dhfr *and *dhps *data the frequency of the highly resistant *dhfr*-*dhps *genotype in each of three time points was estimated. The frequency of this genotype more than quadrupled between 2001 and 2002 in Kilombero/Ulanga and in Rufiji, and it also was found greatly in excess of the numbers expected on the basis of random association. Such population-wide associations between unlinked resistance genes are expected to occur when two drugs with independent modes of action are combined, or where resistance to a single drug is controlled by unlinked mutations. The association occurs because of the combined effects of two selection processes which occur simultaneously. The fully resistant parasites survive better than genotypes with partial resistance. In addition, the drug treatment itself promotes assortative mating among resistant survivors of treatment by purging the co-infecting sensitive genotypes. Recombination, which occurs between gametocytes taken up in the same blood meal, is a force which can disrupt these associations, particularly in high transmission settings and it has been proposed that high recombination might slow the spread of resistance in such areas [51,52]. Models have indicated that recombination can counteract the effect of drug pressure most effectively when the resistance alleles involved are rare, or when drug pressure is low [4,52,53]. The evidence of this study endorses this conclusion since the population-wide association of *dhfr *triple and *dhps *double mutant alleles was not significant when drug pressure was weak but became apparent immediately drug pressure increased. This is a significant consideration in the era of combination treatments for malaria. It suggests that in high transmission settings there may be value in using a suite of anti-malarial drugs for different treatment applications such as intermittent preventive treatment in pregnancy or intermittent preventive treatment of infants rather than blanket use of the same regimen across all malaria treatment applications.

It is widely agreed that malaria treatments should be used in a manner which will minimize drug pressure and limit the growth of resistance. The difficulty lies in gathering empirical support for specific policy alternatives in order to achieve this. Measurements from a variety of different endemic contexts are needed in order to evaluate the effects of policy and drug resistance and the body of evidence from the field is growing [54]. A key observation here is that when resistance alleles are present at low frequency and selection is applied they then spread rapidly. Clearly the first emphasis of policy should always be to minimize the emergence of new mutations and this might be achieved through use of combination therapy. Since this study observed a strong link between government guidelines on treatment practice and drug pressure this is encouraging to the view strategic policy on treatment has the potential to be used in prolonging the useful life of drugs in rural Africa.

## Competing interests

The authors declare that they have no competing interests.

## Authors' contributions

ALM: Participated in study design, carried out the molecular genotyping, statistical analysis, interpretation of the data and drafting of the manuscript. RP: Participated in molecular genotyping, statistical analysis, interpretation of the data and critical review of the manuscript for important intellectual content. SA and PK: Oversaw all aspects of the study, including design and execution of the field work, analysis and interpretation of the data and critical review of the manuscript for important intellectual content. HM: Participated in the conception and designing of the study and critical review of the manuscript for important intellectual content. PB: Original conception and designing of the study and critical review of the manuscript for important intellectual content. CR: Conception of the study, oversaw all the molecular aspect of the study, participated statistical analysis, interpretation of the data and drafting of the manuscript. All the authors read and approve final manuscript for submission

## Supplementary Material

Additional file 1 **Supplementary information**. Observed and expected values for linkage disequilibrium analyses and complete data of haplotype frequencies detected in the study Click here for file
