# Supplementary material for: Drug coverage in treatment of malaria and the consequences for resistance evolution - evidence from the use of sulphadoxine/pyrimethamine
Source: Malar J. 2010 Jul 5;9:190. doi: 10.1186/1475-2875-9-190 (PMC2908640; doi:10.1186/1475-2875-9-190)
Supplement: Additional file 1 — Supplementary information. Observed and expected values for linkage disequilibrium analyses and complete data of haplotype frequencies detected in the study [file 1475-2875-9-190-S1.doc]

**Supplementary information showing the observed and expected values for linkage disequilibrium analyses.**

# A: Two locus genotype tables: frequencies observed/expected

**KILOMBERO/ULANGA 2000**

N=190

|  | SAK | AAK | SGE | Rare alleles |
| --- | --- | --- | --- | --- |
| CNCS | 0.358  0.326 | 0.100  0.100 | 0.037  0.060 | 0.005  0.013 |
| CICN | 0.058  0.048 | 0.000  0.015 | 0.010  0.009 | 0.005  0.002 |
| CNRN | 0.053  0.051 | 0.010  0.016 | 0.016  0.010 | 0.000  0.002 |
| CIRN | 0.179  0.213 | 0.079  0.065 | 0.053  0.039 | 0.016  0.009 |
| Rare alleles | 0.005  0.003 | 0.010  0.001 | 0.005  0.001 | 0.000  0.0002 |

### KILOMBERO/ULANGA 2001

N=148

|  | SAK | AAK | SGE | Rare alleles |
| --- | --- | --- | --- | --- |
| CNCS | 0.351  0.355 | 0.061  0.066 | 0.061  0.059 | 0.013  0.006 |
| CICN | 0.061  0.064 | 0.013  0.012 | 0.013  0.012 | 0.000  0.001 |
| CNRN | 0.108  0.089 | 0.013  0.016 | 0.000  0.015 | 0.000  0.002 |
| CIRN | 0.203  0.217 | 0.047  0.040 | 0.047  0.036 | 0.000  0.004 |
| Rare alleles | 0.007  0.005 | 0.000  0.001 | 0.000  0.001 | 0.000  0.0001 |

**KILOMBERO/ULANGA 2002**

**N**=358

|  | SAK | AAK | SGE | Rare alleles |
| --- | --- | --- | --- | --- |
| CNCS | 0.195  0.151 | 0.022  0.029 | 0.036  0.079 | 0.008  0.004 |
| CICN | 0.064  0.060 | 0.014  0.011 | 0.025  0.031 | 0  0.001 |
| CNRN | 0.053  0.051 | 0.011  0.010 | 0.022  0.027 | 0.003  0.001 |
| CIRN | 0.249  0.302 | 0.059  0.057 | 0.215  0.158 | 0.003  0.007 |
| Rare alleles | 0.014  0.011 | 0.003  0.002 | 0.003  0.006 | 0  0.0003 |

**RUFIJI 2000**

N=285

|  | SAK | AAK | SGE | Rare alleles |
| --- | --- | --- | --- | --- |
| CNCS | 0.263  0.256 | 0.060  0.055 | 0.017  0.028 | 0.007  0.008 |
| CICN | 0.098  0.093 | 0.025  0.020 | 0.000  0.010 | 0.003  0.003 |
| CNRN | 0.081  0.096 | 0.032  0.020 | 0.014  0.010 | 0.003  0.003 |
| CIRN | 0.270  0.264 | 0.035  0.056 | 0.042  0.029 | 0.010  0.009 |
| Rare alleles | 0.025  0.028 | 0.007  0.006 | 0.007  0.003 | 0.000  0.001 |

**RUFIJI 2001**

N=282

|  | SAK | AAK | SGE | Rare alleles |
| --- | --- | --- | --- | --- |
| CNCS | 0.209  0.218 | 0.057  0.040 | 0.025  0.036 | 0.007  0.004 |
| CICN | 0.074  0.070 | 0.007  0.013 | 0.014  0.012 | 0.000  0.001 |
| CNRN | 0.082  0.078 | 0.011  0.014 | 0.011  0.013 | 0.003  0.001 |
| CIRN | 0.351  0.352 | 0.060  0.065 | 0.067  0.058 | 0.003  0.007 |
| Rare alleles | 0.014  0.013 | 0.000  0.002 | 0.003  0.002 | 0.000  0.0002 |

## RUFIJI 2002

N=342

|  | SAK | AAK | SGE | Rare alleles |
| --- | --- | --- | --- | --- |
| CNCS | 0.108  0.086 | 0.032  0.021 | 0.020  0.050 | 0.003  0.007 |
| CICN | 0.053  0.049 | 0.015  0.012 | 0.023  0.028 | 0.003  0.004 |
| CNRN | 0.047  0.046 | 0.015  0.011 | 0.023  0.027 | 0.003  0.004 |
| CIRN | 0.313  0.340 | 0.061  0.081 | 0.237  0.196 | 0.035  0.026 |
| Rare alleles | 0.006  0.003 | 0.003  0.001 | 0.000  0.002 | 0.000  0.0003 |

**=significance p≤0.001**

|  |
| --- |

**B: *Dhfr* and *dhps* haplotype frequences**

| **2000**  **Haplotype** | **KILOMBERO/ULANGA** | | **RUFIJI** | |
| --- | --- | --- | --- | --- |
|  | **N = 365** | **Freq** | **N = 417** | **Freq** |
| SAKAA | 251 | 0.6877 | 297 | 0.7122 |
| AAKAA | 61 | 0.1671 | 74 | 0.1775 |
| SGEAA | 46 | 0.1260 | 33 | 0.0791 |
| CAKAA | 2 | 0.0055 | 2 | 0.0048 |
| FAKAA | 3 | 0.0082 | 5 | 0.0120 |
| SGKAA | 1 | 0.0027 | 3 | 0.0072 |
| SAEAA | 1 | 0.0027 | 1 | 0.0024 |
| AAEAA | 0 | - | 2 | 0.0048 |

| **2000**  **Haplotype** | **KILOMBERO/ULANGA** | | **RUFIJI** | |
| --- | --- | --- | --- | --- |
|  | **N = 376** | **Freq** | **N =455** | **Freq** |
| CNCSVI | 193 | 0.51 | 162 | 0.36 |
| CIRNVI | 105 | 0.28 | 171 | 0.38 |
| CNRNVI | 28 | 0.075 | 53 | 0.12 |
| CICNVI | 40 | 0.11 | 51 | 0.11 |
| CNCNVI | 7 | 0.019 | 5 | 0.01 |
| CNRSVI | 2 | 0.005 | 7 | 0.02 |
| CIRSVI | 1 | 0.003 | 4 | 0.01 |
| CICSVI | 0 | 0 | 2 | 0.004 |

| **2001**  **Haplotype** | **KILOMBERO/ULANGA** | | **RUFIJI** | |
| --- | --- | --- | --- | --- |
|  | **N=294** | **Freq** | **N=519** | **Freq** |
| SAKAA | 205 | 0.697 | 400 | 0.7707 |
| AAKAA | 52 | 0.177 | 58 | 0.1117 |
| SGEAA | 32 | 0.109 | 49 | 0.0944 |
| CAKAA | 3 | 0.0102 | 2 | 0.0038 |
| FAKAA | 2 | 0.0034 | 10 | 0.0193 |

| **2001**  **Haplotype** | **KILOMBERO/ULANGA** | | **RUFIJI** | |
| --- | --- | --- | --- | --- |
|  | **N=238** | **Freq** | **N=420** | **Freq** |
| CNCSVI | 113 | 0.4748 | 127 | 0.3024 |
| CIRNVI | 74 | 0.3109 | 196 | 0.4667 |
| CNRNVI | 27 | 0.1134 | 46 | 0.1095 |
| CICNVI | 23 | 0.0966 | 43 | 0.1024 |
| CNCNVI | 1 | 0.0042 | 8 | 0.0190 |

| **2002**  **Haplotype** | **KILOMBERO/ULANGA** | | **RUFIJI** | |
| --- | --- | --- | --- | --- |
|  | **N=603** | **Freq** | **N=596** | **Freq** |
| SAKAA | 359 | 0.5954 | 357 | 0.5990 |
| AAKAA | 75 | 0.1244 | 78 | 0.1309 |
| SGEAA | 165 | 0.2736 | 146 | 0.2450 |
| CAKAA | 3 | 0.0050 | 4 | 0.0067 |
| FAKAA | 0 | - | 8 | 0.0134 |
| SGKAA | 0 | - | 3 | 0.0050 |
| SAEAA | 1 | 0.0017 | 0 | 0 |

| **2002**  **Haplotype** | **KILOMBERO/ULANGA** | | **RUFIJI** | |
| --- | --- | --- | --- | --- |
|  | **N=489** | **Freq** | **N=527** | **Freq** |
| CNCSVI | 136 | 0.2781 | 97 | 0.1841 |
| CIRNVI | 252 | 0.5153 | 330 | 0.6262 |
| CNRNVI | 45 | 0.0920 | 44 | 0.0835 |
| CICNVI | 47 | 0.0961 | 48 | 0.0911 |
| CNCNVI | 4 | 0.0082 | 8 | 0.0152 |
| CNRSVI | 4 | 0.0082 | 0 | - |
| CIRSVI | 1 | 0.0020 | 0 | - |
